# *Fusobacterium* and Colorectal Cancer

**DOI:** 10.3389/fonc.2018.00371

**Published:** 2018-10-15

**Authors:** Ziwei Zhou, Jiewen Chen, Herui Yao, Hai Hu

**Affiliations:** ^1^Department of Oncology, Sun Yat-Sen Memorial Hospital, Sun Yat-Sen University, Guangzhou, China; ^2^Breast Tumor Center, Sun Yat-Sen Memorial Hospital, Sun Yat-Sen University, Guangzhou, China

**Keywords:** *Fusobacterium*, colorectal cancer, tumor microenvironment, epigenetic changes, tumor immunity

## Abstract

Colorectal cancer (CRC) is the third most common cancer worldwide and its pathogenesis has been extensively explored over the past decades. Recently, microorganisms in the gastrointestinal tract have emerged as potential etiological agents. In particular, a direct proportional association between *Fusobacterium* and CRC has been described. Since then, the functional impact of *Fusobacterium* in CRC development has been studied using various mouse models. Although some epidemiologic studies did not establish an obvious relationship between *Fusobacterium* and CRC, numerous pathogenic mechanisms leading to the disease have been described. For instance, *Fusobacterium* can activate the E-cadherin/β-catenin signaling pathway and is associated with particular epigenetic phenotype, such as microsatellite instability (MSI) and hypermethylation, via its strong adhesive and invasive abilities resulting in malignant transformation of epithelial cells. Also, *Fusobacterium* could alter the tumor microenvironment (TME) significantly by myeloid-derived suppressor cells (MDSCs), tumor associated macrophages (TAMs), and tumor associated neutrophils (TANs) recruitment and local immune suppression. Herein, we provide an in-depth review of the relationship between *Fusobacterium* and colorectal cancer. In light of the emergence of microbiome-based therapeutics, potential therapies and preventive strategies for colorectal cancer related to *Fusobacterium* are also discussed.

## Introduction

Commensal bacteria in the colon might play a significant role in the maintenance of health (1–4). Intestinal microbiota promotes the maturation of human immune system and maintenance of natural barrier integrity (5). Bacterial dysbiosis in the gut has been associated with numerous human diseases, including obesity (6, 7), intestinal diseases (8, 9), cardiovascular diseases (10), autism (11), malignancies (12, 13, 14) and others. Garrett *et al*. identified that colitis could be transferred from *T-bet*^−/−^ × *Rag2*^−/−^ ulcerative colitis (TRUC) mice to wild type mice with particular intestinal bacteria (*Proteus mirabilis* and *Klebsiella pneumoniae*) transplantation (15). The metabolic phenotypes and adiposity status could also be modulated by cultured gut microbiota from human and coordinate diets in mice (6). This finding denotes the importance of gut microbiota symbiosis and the dysfunctional proportion could exacerbate human diseases, since both normal and pathogenic flora are important in the regulation of homeostasis (16). Particularly, colonic dysbiosis has been associated with CRC (17) (12, 18) (19) (20). Among the multitudinous genera, *Fusobacterium* stood out as being oftentimes increased in CRC (14, 21) (Table 1). As in the case of *Helicobacter Pylori* correlation with gastric cancer, *Fusobacterium* may be an essential microbial carcinogen that fuels the initiation and development of CRC (21, 22). *Fusobacterium* is a genus of gram-negative anaerobic bacteria. It may act as a main anchor of biofilms that can induce periodontitis (23, 24), vaginitis (25) and other infections (26). *Fusobacterium* was considered as part of the normal flora of the oropharynx formerly, but lately its pathogenic role especially as a driver of periodontitis (27) and its association with intestinal diseases has been demonstrated. Although it is still unclear whether *Fusobacterium* is the passenger or driver of CRC, many studies have concluded that *Fusobacterium* is a novel risk factor for CRC development and progression, as well as a determinant affecting patient survival outcomes (13, 28, 29). Kostic *et al*. (14) illustrated that in colorectal adenoma, an early event in CRC development, *Fusobacterium* is found to be enriched in comparison with surrounding normal tissue suggesting an essential role of *Fusobacterium* in the early onset of CRC. Moreover, a recent retrospective study with 13,096 adult patients suggested that those presented with *Fusobacterium nucleatum* (one of the species of *Fusobacterium*) have increased risk of CRC (30). Additionally, significantly larger proportions of *Fusobacterium* has been detected from feces of adenoma and CRC patients in comparison to healthy controls, which further confirms Kostic's finding. Distal metastasis of colonic cancer was also found to be colonized with *Fusobacterium* and other assembled microbes. Investigators have also shown that tumor proliferation and cancer growth could be reduced via decreasing the load of *Fusobacterium* by antibiotic treatment (metronidazole) (31). Moreover, *Fusobacterium* is associated with certain epigenetic phenotypes of CRC –high degrees of microsatellite instability (MSI) and CpG island methylation phenotype (CIMP) (32, 33), which could provide promising opportunities to develop diagnostic tools or treatment biomarkers for CRC.

**Table 1:** Studies with positive detection of *Fusobacterium* in colorectal diseases.

**Authors**	**Diseases**	**Methods**	**Samples**
McCoy et al. (34)	Adenoma	qPCR, FISH	Human tissues
Wong et al. (35)	Adenoma	qPCR	Feces
Ito et al. (36)	Adenoma	qPCR	FFPE tissues
Kostic et al.(21)	CRC	RNA-seq, qPCR, WGS	Human tissues
Mima et al. (37)	CRC	qPCR	FFPE tissues
Kostic et al. (14)	Adenoma and CRC	qPCR, 16S rDNA Sequence, WAS, FISH	Human tissues, Feces
Tahara et al. (33)	CRC	qPCR	Human tissues
Wang et al. (38)	CRC	ELISA, WB, qPCR	blood samples and feces
Mehta et al. (29)	CRC	qPCR	FFPE tissues

*qPCR, quantitative real-time polymerase chain reaction; FISH, Fluorescent quantitative polymerase chain reaction; RNA seq, RNA sequencing; WAS, Whole-genome sequence; ELISA, Enzyme-linked immunosorbent assay; WB, Western blotting; FFPE, Formalin-fixed paraffin-embedded*.

## The biological features of *fusobacterium*

### A heterogeneous genus of bacteria

*Fusobacterium* is a cylindrical shaped, gram-negative, non-spore-forming, strictly anaerobic genus. Although *Fusobacterium* is part of the normal microbiome, recent findings indicated that increased *Fusobacterium* levels have been detected in various inflammatory (39–41) and cancer samples (33). There are 14 species in *Fusobacterium*, such as *F. necrophorum* (inhabitant of the alimentary tract and being responsible for Lemierre' syndrome) and *F. varium* (found in the ulcerative colitis). Among them, *F. nucleatum* is one of the key pathogens which plays a role in oral plaque formation, due to its adhesive ability, serving as a bridge organism during colonization and biofilm formation (42). Although several studies suggest that *Fusobacterium* strains might vary in their virulence potential, it is has been speculated that some *Fusobacterium* strains can acquire genes through horizontal transfer and obtain increased virulence potential from different species and strains (43, 44, 45). Regardless of the mechanism in which *Fusobacterium* attains its virulence, evidence points to the positive correlation of this *Fusobacterium* toward CRC malignancy.

### Adhesion and invasion of *fusobacterium* into host tissue cells

*Fusobacterium* is an invasive organism. *Fusobacterium* invades host with the aid of a surface adhesion molecule called FadA, which is abundantly expressed on *Fusobacterium* (46). Rubinstein and his colleagues suggested that FadA, a membrane protein, encoded by *Fusobacterium* binds to E-cadherin on the epithelial cell surface and leads to E-cadherin phosphorylation and internalization. This, in turn, activates β-catenin signaling pathways (22) and consequently leads to inflammation and tumorigenesis gene transcription (such as NF-κB, Myc ad Cyclin D1, lymphoid enhance factor/T cell factor). Furthermore, they detected higher FadA expression levels in CRC tissue compared with normal tissue. Consistently, the expression of oncogenes, inflammatory genes and *Wnt* genes are increased in CRC cells under modulation of purified FadA. Further experiments with purified FadA or *F. nucleatum* could only stimulated cell lines with APC or β-catenin mutations but not non-cancerous HEK-293 cells, indicated that oncogenesis promoted by *Fusobacterium* was secondary to these significant mutation events. As an invasive organism that survives inside host cells, *Fusobacterium* is also capable of releasing RNA into the host cell cytoplasm that is detected by cytosolic retinoic acid-inducible gene 1 (RIG-1), triggering activation of NF-κB and activating inflammatory genes and oncogenes (47, 48). Furthermore, FadA binds to vascular endothelial cadherin (VE-cadherin), a cell-cell junction molecule identified as the endothelial receptor for FadA. FadA binding causes VE-cadherin to relocate and increases the endothelial permeability, which then facilitates *Fusobacterium* and other bacteria species to penetrate into the blood stream (49). *Fusobacterium* also has a strong ability to induce co-aggregation and shuttle unrelated microbes [in particular *Streptococcus* and *Campylobacter* (50)] into host cell via Fap2, a large membrane protein present in *Fusobacterium* The shuttled microbes are known to have weak binding ability to host cells or they are non-invasive (51, 52). Being shuttled into the host cells by *Fusobacterium*, the toxic effects on endothelial cells become multifold.

### Microbial–host interaction and inter-microbial interaction

Normally, the interaction between intestinal microbiota and the host contributes to the maintenance of homeostasis and normal mucosal immunity. Abnormalities on either side will disrupt this balance and brings about disease and malignancy. It has already been established that lesions on the intestinal mucosa impair barrier function (53). In tumor microenvironment (TME), the pH value of the tumor tissue is around 6.5-6.9, which is slightly lower than the normal physicological pH of 7.4 (51, 52), This slight change of *pH* can significantly affect the refined composition of microbial community in TME (54). In *APC* gene deleted mouse models (CRC animal models), significant defective intestinal barrier function at tumor sites has been described and therefore concluded that changes in the local environment and a deficient barrier could provide favorable condition for *Fusobacterium* to reproduce and cause further mucosal injury (55). Hence, it is hypothesized that the growth of *Fusobacterium*, normally a commensal at low abundance, is increased with disruption of intestinal homeostasis and in turn accelerates tumorigenesis, consequently forming a vicious positive feedback cycle.

However, as only one of the estimated 10^3^ microbial species in the colon (56), *Fusobacterium* cannot be studied in isolation and some studies have identified other bacterial species that may also contribute to colorectal carcinogenesis and have interaction with *Fusobacterium*. These bacteria include and *Escherichia coli* (*E. coli*) (20), *Bacteroides fragilis* (*B. fragilis*) (57), *H. pylori* (58), *Enterococcus faecalis* (*E.faecalis*) (59), *Streptococcus bovis* (*S. bovis*) (60) and *Clostridium septicum* (*C. septicum*) (61). Moreover, as an oral commensal and a periodontal pathogen, *Fusobacterium* also interacts with *candida albicans* within the oral cavity (62). As evidenced by other oral community members (such as members of the *Porphyromonas*) that has been found on colonic tumors (63), *Fusobacterium* may also play a role in the link between oral and intestinal microbiota. *Fusobacterium* has defense ability against human neutrophilic peptide-1 (64) and is one of the most important anaerobes that promote the formation of dental biofilms (24, 65). The polymicrobial nature of oral biofilms and the asaccharolytic metabolism of many of these species allows them to survive comfortably in the microenvironment of colonic lesions (66). Consequently, co-occurrence patterns of *Fusobacterium* with other microbiota in the oral cavity may correlate with localized intestinal dysbiosis in the setting of colorectal carcinogenesis.

## The impact of *fusobacterium* in CRC microenvironment

**Figure 1:**
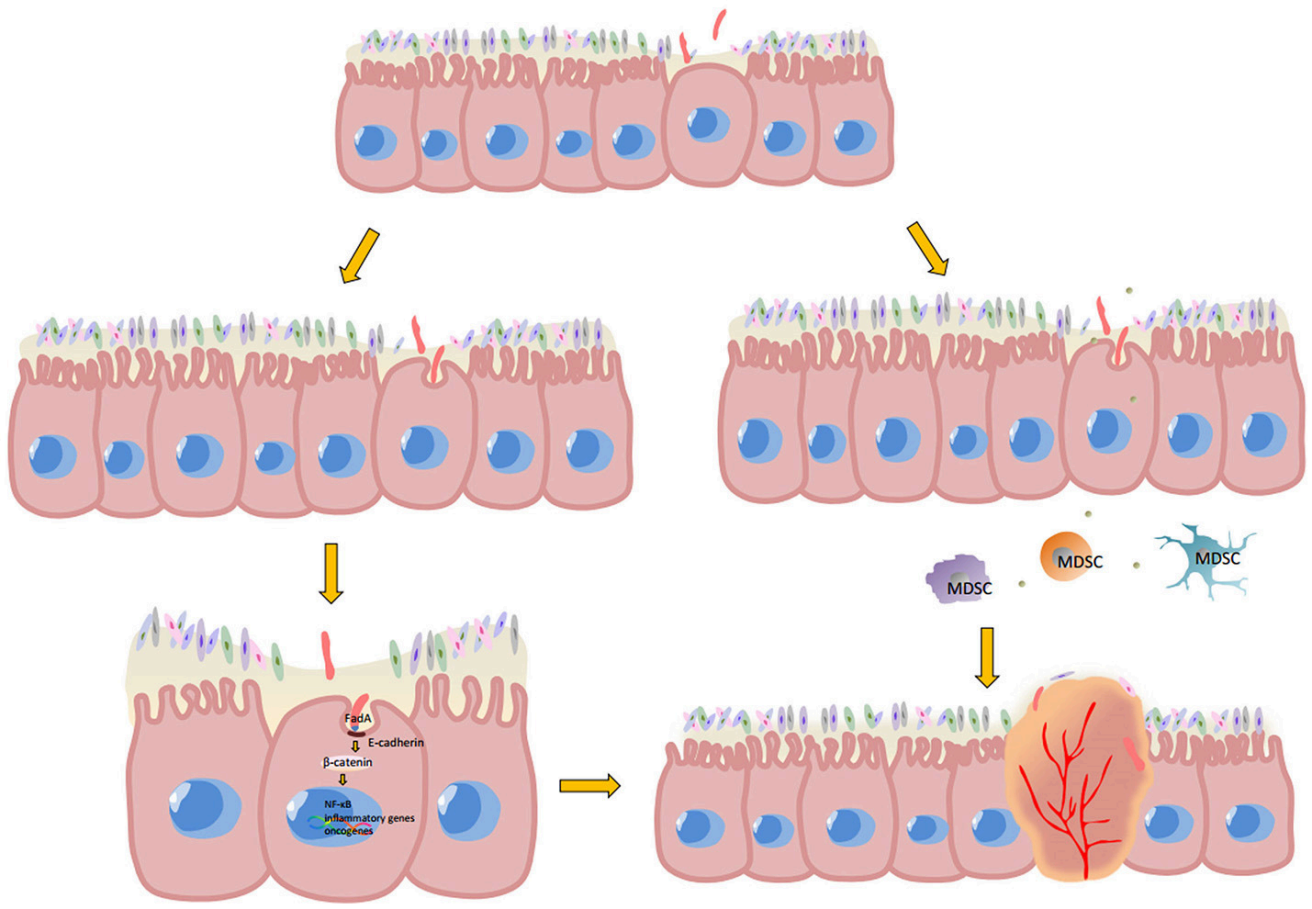
Potential mechanism of *Fusobacterium*-associated CRC. Mutant epithelial cells cause local intestinal barrier impairment, which gives *Fusobacterium* the opportunity for adherence and subsequent invasion into epithelial cells. Once FadA, a membrane protein of *Fusobacterium*, combines with E-cadherin and is internalized by epithelial cells, the β-catenin signaling pathway is activated. Phosphorylated β-catenin would enter the cell nucleus from cytoplasm and promote NF-κB genes, pro-inflammatory genes and the expression of many other oncogenes. Moreover, the microenvironment of malignancies is anoxic and acidic, which would be more suitable for *Fusobacterium* reproduction compared to other bacteria. The metabolites of aggregated *Fusobacterium*. Then recruit MDSCs, in turn suppressing anti-tumor immunity and promote CRC carcinogenesis.

Sporadic colorectal cancer and colitis-associated colorectal cancer are predominant classes of CRC. Sporadic colorectal cancer develops in five stages—polyp, early adenoma, late adenoma, carcinoma and invasion; while colitis-associated cancer develops in six stages—colitis, indefinite dysplasia, low-grade dysplasia, high-grade dysplasia, carcinoma and invasion (67). In both cases, *Fusobacterium* was found to be an important associated factor in their development (34, 68, 69). Oncogenesis caused by *Fusobacterium* is closely related to the inflammatory state of the tumor. However, the crosstalk between each inflammatory component of the tumor microenvironment is very complicated. For example, the abundance of tumor infiltrating lymphocytes (TILs) correlate with improved clinical outcomes in colorectal patients (70–72), while the presence of myeloid derived suppressive cells (MDSCs) (73) and regulatory T cells (Tregs) (74) indicate immune inhibitory status and are associated with poor prognosis. Hereby, the detailed mechanism of the interaction of *Fusobacterium* with each TME component in inflammatory and malignancy state is discussed as follows (Figure 1).

### *Fusobacterium* suppresses the immune response

Kostic et al. (21) found that CD11b+ myeloid derived suppressive cells (MDSCs), including granulocytes/tumor associated neutrophils (TANs) and macrophages/tumor associated macrophages (TAMs), are more abundant in tumor tissues than normal tissue of *Fusobacterium*-fed APC Min mice. MDSCs are a heterogeneous group of immune cells differentiating from the myeloid lineage (21, 75). MDSCs are highly abundant in some pathological situations such as chronic infections and cancer. Due to their immunosuppressive activities and interaction with other immune cell types, tumor tissue with high infiltration of MDSCs may predict poor prognosis and drug resistance (76, 77). TANs and TAMs, the special subset of MDSCs, also contribute to the inhibition of anti-tumor immunity and result in tumor progression and metastasis, which has been verified in considerable experimental data from clinical and pre-clinical studies (48, 56).

In mouse models, MDSCs express both myeloid lineage differentiation antigen Gr1 (granulocyte differentiation antigen1) and CD11b and are further classified into two subtypes—monocytic and granulocytic (78–80). *F. nucleatum* increases the production of reactive oxygen species (ROS) (81) and inflammatory cytokines such as IL-10 in CRC (34), possibly by recruiting MDSCs. ROS normally serve as second messengers to regulate many of the intracellular signaling cascades that govern multiple cellular activities (81), which causes DNA damage and other cell injuries. It is well documented that inflammatory oxidative stress can lead to *p53* mutation, thus promoting oncogenesis (82). Oxidative stress also contributes to aberrant hypermethylation (83), which in turn causes tumor-suppressive gene inactivation and carcinogenesis (84, 85). Hypermethylation could also occur in promoter regions of mismatch repair protein (MLH1) gene and result in microsatellite instability (MSI) (86–88), which was recognized as an early molecular phenomenon in CRC. Moreover, MDSCs can disrupt the anti-tumor abilities of the immune system by secreting arginase-1 (89), which suppresses T cell activity and induces tumor intolerance (90, 91).

MDSCs also interact with other immune cell types, such as an anti-tumor subtype of dendritic cells (CD103+ DCs). Mice treated with *Fusobacterium* were found to have more CD103+ DCs within tumors compared with control groups (21). These cells regulate immune responses by promoting the activation of Foxp3^+^ regulatory T cells, a CD4^+^ T cell subset that inhibits cytotoxic and effector T cells, and results in restrained antitumor immunity (92). In addition, immunohistochemical analysis indicate that a higher amount of *Fusobacterium* were associated with a lower density of tumor inhibiting CD3+ T cells in tumor tissue, thereby enabling tumor to escape immune surveillance (37). Additionally, *Fusobacterium* could suppress immune function by arresting T cells in the Mid-G1 phase of the cell cycle. Shenker and Datar discovered that *Fusobacterium* suspended T cell proliferation at Mid-G1 phase by regulating different cyclin levels via an immunosuppressive protein, *F. nucleatum* immunosuppressive protein (FIP) (93). They also found that proliferating cell nuclear antigen (PCNA) was significantly decreased in expression. PCNA is a DNA clamp that acts as a processivity factor for DNA polymerase δ and accelerates cell transition through early to mid-G1. With decreased proliferative ability, T cell would fail to function or attack cancer cells thus progressing to an immunosuppressive phase.

Fap2, an outer membrane protein of *Fusobacterium*, is also involved in tumor suppression activity. Kaplan found that Fap2, together with adhesin RadD can cause lymphocyte death by direct contact with target lymphocytes. Either Fap2 or RadD alone could induce lymphocyte death, though at a much lower level. Chamutal Gur also discovered that Fap2 could bind to TIGIT, an inhibitory receptor presents on all human NK cells and various T cells, protecting tumors from immune attack by inhibiting effective immune cell activity (94).

### *Fusobacterium* promotes development from inflammation to malignancy

Recent findings support the claim that *Fusobacterium* could enhance the development from being in inflammatory state to malignancy. This finding was first reported by Kostic (21) and colleagues when *APC*^*Min*/+^ mice were introduced to human isolates of *Fusobacterium nucleatum*. Higher level of inflammation and more colonic tumors were found in the *F. nucleatum* group compared to control. However the colitis mice model (IL-10^−/−^ and Rag2^−/−^/T-bet^−/−^) treated with *F. nucleatum* did not develop colonic tumors. This may suggest that *Fusobacterium* induce oncogenesis downstream of the *APC* pathway and the tumorigenesis does not depend on pre-existing colitis condition because the colitis mice did not develop colon tumors after *F. nucleatum* introduction. They also revealed the expression signature of inflammation: the higher expression of COX-2, IL-1 β, IL-6, IL-8, TNF, and MMP3 by human and mouse cell line while co-culturing with *Fusobacterium* suggests an NF-κB -driven pro-inflammatory response. Rubinstein et al. further indicated that the elevated expression of inflammatory genes such as NF-κB, IL-6, IL-8 and IL-18 were correlated with FadA level in CRC tissues, which is consistent with the former study. Additionally, *Fusobacterium* also binds to Toll-like receptor 4 (TLR4) of epithelial cells and activate the TLR4/MYD88/NF-κB signaling pathway. NF-κB p50/p65 separated with phosphorylated IκB and bound to DNA, followed with microRNA-21 transcription. microR-21 could inhibit RAS p21 GTPase activating protein (RASA1) and therefore activate the RAS/RAF/MEK/ERK signaling pathway (68). Thus, in the tumor-initiation period, *Fusobacterium* exerts its tumor-promoting action through the augmentation of local inflammation. Furthermore, some indicative molecules in human colon samples, such as COX-2, IL-1β, IL-6, IL-8, TNF-α, and MMP3, are suggestive of activation of NK-κB-driven inflammation (21, 55, 95). Among these expression signatures, IL-8, TNF-α and other chemokines could recruit neutrophils and macrophages, which synthesize nitric oxide (NO) and cause oxidative stress to epithelial and stromal cells. This results in DNA damage and consequently activation of *p53* transcription which in turn suppresses tumorigenesis by inducing G1-S arrest, DNA repair and cell apoptosis. Moreover, *p53* overexpression also leads to *TP53* mutation, which is a key event during CRC development. Additionally, chronic inflammation and ROS production cause many other mutations (such as CHD7 and CHD8, members of the chromodomain helicase/ATP-dependent chromatin remodeling family (33, 96) and genomic instability), all of which would accelerate CRC development.

## *Fusobacterium* induces epigenetic changes in tumor cells

Intriguingly, high load of *Fusobacterium* was recently reported to be associated with a specific epigenetic phenotype of CRC. *Fusobacterium* detected in colorectal cancer tissues was related to CpG island methylator phenotype (CIMP) status, high MSI and MLH1 hypermethylation (33) and up-regulating expression of microRNA-21. Interestingly, these specific molecular features of colorectal cancer occur mostly in the ascending colon (97, 98), which is also the most common colonization site of *Fusobacterium* in the GI tract. This might indicate some association between *Fusobacterium* biogeography and the colonic mucosal microenvironment.

CIMP is characterized by simultaneous hypermethylation of numerous CpG islands surrounding the promoter regions of several genes. The high level of methylation of CpG island indicates chronic inflammation and an aggravated immune response (99). Microsatellite instability is the somatic accumulation of length variations in repetitive DNA sequences (33). It has been established that defects in the DNA mismatch repair (MMR) pathway lead to disincorporation, insertions and deletions in microsatellite in this repetitive DNA sequences. MSI is frequently observed in both hereditary and sporadic CRC (100). Inflammatory state and reactive oxygen stress produced by *Fusobacterium* may contribute to epigenetic silencing of the MMR protein MLH1 and reduction of its enzymatic activity, which leads to MSI CRC (101).

microRNAs, a small non-coding RNA functioning in RNA silence and regulating post-transcriptional gene expression, rapidly emerging as promising diagnostic and therapeutic targets, may be involved in the progression of cancer as well (102). Studies suggest that *Fusobacterium* might raise the level of microRNA-21 in tumor cells via epigenetic regulation during macrophage inflammatory response (68). microRNA-21 in turn increases the levels of IL-10 and PGE2 (prostaglandin E2), which inhibit antitumor immunity mediated by T cells in the TME (103, 104), and therefore high level of microRNA-21 usually indicates worse clinical outcomes (105). Nevertheless, further studies are needed in order to pinpoint the relationship between *Fusobacterium* and microRNAs and its significance.

## Potential management of *fusobacterium*-associated CRC

Numerous studies have found that enrichment of *Fusobacterium* is related to worse clinical outcome in CRC patients (96). Analogous to *Helicobacter Pylori* in the setting of gastric cancer, *Fusobacterium* may be an essential pathogen that fuels the initiation and development of CRC. Research has suggested that reducing the abundance of *Fusobacterium* may help patients with intestinal diseases such as IBD to recover (106). Therefore, therapies that specifically target *Fusobacterium* or their mechanism of action could be developed for the prevention or treatment of CRC.

### Reducing carcinogenicity of *fusobacterium*

As we discussed early, FadA, a membrane protein of *Fusobacterium*, induces adhesion and invasion, and is the originator of epithelial injury. In FadA-E cadherin pathway, FadA gene copy number has been found to have a direct correlation with either healthy, pre-cancerous or CRC states (22), suggesting that FadA may be a promising biomarker in CRC diagnosis. Targeting FadA blockade development is also promising by inhibiting its adhesive and invasive abilities. As E-cadherin internalization via clathrin is a key process during FadA/E-cadherin/β-catenin pathway, a clathrin inhibitor (Pitstop2) could block the pro-carcinogenesis pathway (22). Similarly, Fap2, which ferries other bacteria into the host cell, is also a membrane protein of *Fusobacterium* The hemagglutination and coaggregation function of *Fusobacterium* can be inhibited by galactose (52). This indicates that membrane blockers may suppress key pathogenic features of *Fusobacterium*, providing a further target in the early stages of *Fusobacterium* pathogenesis. However, the possibility should be taken into consideration that abundance of FadA might only reflect *Fusobacterium* infection status at a given time point. Since *E. coli* and *Streptococcus gallyliticus* (107) and many other microorganisms could also be significant pathogenesis factors and promote oncogenesis via different mechanisms, FadA alone may not be sufficient. Moreover, *Fusobacterium* is a heterogeneous genus and further gene and protein differences between low-virulence strains and high-virulence strains will need to be probed by genome and proteome analysis (108). Such approaches may allow for characterizing relevant species at a much more granular level and identifying those equipped with highly oncogenic motifs.

Studies have found that some *F. nucleatum* strains may acquire genes through horizontal transfer, indicating the close connection between *Fusobacterium* and other gut microbes (44, 45). This can be seen in the therapeutic effects of microecologic products (109), such as probiotics and prebiotics, as they reduce the overall virulence genera through regulating the whole composition of microbes and cut down the possibility for *Fusobacterium* to obtain virulence genes. For example, the amount of *Fusobacterium* was reduced in fecal samples and the microbial diversity increased following probiotic intervention in CRC patients, suggesting that microecologic products would benefit CRC patient for suppressing CRC-associated genera (110). Though limited literature has proved a direct relationship between *Fusobacterium* colonized in gingival sulcus and colorectal cancer tumorigenesis, there might be some connections between oral flora and gut flora, as well as connections between oral infectious diseases and intestinal diseases (such as CRC). Combined detection of fecal and oral microbes may also enhance prediction of adenomas or CRC in addition to other risk factors (111). It is possible that altering intestinal Fusobacterium abundance could be approached by targeting oral *Fusobacterium*, however further studies are needed for confirmation.

### Tackling drug resistance and dealing with a complicated microenvironment

As stated earlier, *Fusobacterium* has a complex influence on the tumor microenvironment. Either chemotherapy or targeted therapies for CRC face the serious dilemma of drug resistance (112, 113). Compared with other tumors, CRC uniquely occurs in an intricate microenvironment owing to the co-existence of the huge diversity of microorganisms within the gut microbiome. It is now understood that drug resistance is closely related to the tumor microenvironment (114, 115). In this sense, the microenvironment regulated by *Fusobacterium* might account for drug resistance in CRC. On the one hand, *Fusobacterium nucleatum* has been found to promote chemoresistance to colorectal cancer by modulating autophagy (116). Via binding to TLR4 on colorectal cancer cells, *Fusobacterium nucleatum* activated the TLR4/MYD88 innate immune signaling pathway and miRNA-18a and miRNA-4802 were downregulated sequentially, which resulted in ULK1 and ATK7 expression. Both ULK1 and ATG7 are important elements of autophagy, the downregulation of which could decrease CRC cell apoptosis induced by chemotherapy agents. On the other hand, *Fusobacterium* might contribute to anti-VEGF pathway agents, such as bevacizumab. *Fusobacterium* promotes inflammation and cause aberrant extracellular matrix (ECM) formation, which accelerate VEGF synthesis and secretion. Many of the *Fusobacterium* –associated genes, including IL-6 and IL-8, also play significant roles in alternative pro-angiogenesis pathways (117). Besides, MDSCs recruited by *Fusobacterium* are also associated with drug resistance (118, 119). With overactive inflammation-tumorigenesis sequences and a local immunosuppressive state, it is more likely for patients to undergo drug resistance to immune therapies. Facing treatment failure, strategies to lower the abundance of *Fusobacterium* may be helpful to reduce drug resistance.

### Exploring the relationship between *fusobacterium* and epigenetic alterations

Some studies implicate *Fusobacterium* in certain epigenetic changes of CRC, such as CpG island methylation phenotype (CIMP), MSI and microRNA expression. However, further exploration is needed to uncover mechanism of how *Fusobacterium* impacts on CRC epigenetic changes. Particularly, CRC tissues enriched with *Fusobacterium* may be associated with MSI (96). Interestingly, high-abundance of *Fusobacterium* suggests poor prognosis of CRC while MSI forecasts favorable prognosis. One possible explanation is that MSI is not a predominant factor in the interaction between *Fusobacterium* and host cells, and other factors should also be taken into consideration. Recently, MSI is regarded as a strong biomarker for PD-1 blockade (120). It would be a possible explanation that CRC cells with frameshift mutations in the absence of normal mismatch repair function produce a mutation-associated neoantigen, which may activate anti-tumor immunity and enhance the effect of PD-L1 blockade. Some studies have indicated the relationship between gut microbiota and checkpoint immunotherapy recently (121–124), but whether *Fusobacterium* impact on checkpoint inhibitors still remains unclear. Further studies are needed to clarify whether *Fusobacterium* influences mismatch repair signaling pathways and alters the patient's immune response.

## Future outlook and caveats

Colorectal cancer is one of the most common cancers worldwide and an understanding of its relationship with the gut microbiota is beginning to emerge. Interacting with numerous microbes, the initiation and development of colorectal cancer may be fueled by pathogens or inhibited by probiotics. Researchers have demonstrated the correlation between *Fusobacterium* with colonic adenomas and colorectal cancer. Whether this is a cause or just an association with CRC remains unclear, although some potential mechanisms have been elucidated. While FadA-induced cell invasion and subsequent signaling pathway activation is one of the most accepted mechanisms, nevertheless, based on the evidence so far, screening or risk stratification strategies utilizing *Fusobacterium* should be developed. In accordance with the broad public acceptance of probiotics and prebiotics, microbiome-targeted therapies for CRC are a promising avenue for future development. Notably, fecal microbiota transplantation is now considered standard of care for recurrent *Clostridium difficile* infection, with promising results in other conditions reported (125, 126). However, not all of the study results are in agreement with a “causal relationship” conclusion: Dejea et al. found that no consistent bacterial genus associated with tumors by high-throughput sequencing in 30 CRC and 6 adenoma human samples (127). In another paper, Dejea and colleagues studied patchy biofilms in colonic mucosa of patients with an *APC* gene mutations (128). *Fusobacterium* was not the predominant component of the biofilms while *Escherichia coli* and *Bacteroides fragilis* were prominently associated. Moreover, although *Fusobacterium* was more abundant in CRC biopsies and fecal samples than normal control group (129), the elevated level was only manifested in 20–30% of CRC patients and has not been consistent. In a cohort study of 137217 adults (29) to explore the association between diets with CRC prevalence, a stronger relationship between diet and CRC was found in *F. nucleatum* enriched individuals than subgroups without *F. nucleatum* detection. This finding also suggests that *Fusobacterium* may play different roles in different subtypes of CRC. Additionally, the interaction between the microbiota and carcinogenesis in the colon is sophisticated. Studies have also found associations with other microorganisms triggering neoplasia as well as associated mechanisms. For instance, *Enterotoxigenic Bacteoides fragilis* promotes Th17 development by Tregs, limiting the availability of IL2 in the local microenvironment (130) while *Escherichia coli* releases colibactin, a genomic product of polyketide synthase island, which is carcinogenic and promotes CRC development (20).

## Conclusion

In conclusion, how *Fusobacterium* impacts CRC warrants large-scale cohort studies and laboratory experiments. Some key studies suggest that *Fusobacterium* could activate the β-catenin signaling pathway to promote oncogene transcription and alter the tumor microenvironment to induce immune suppression, while others suggested that *Fusobacterium* is associated with epigenetic changes of malignant epithelial cells. Potential microbiome therapies in colorectal cancer are still some time away but are likely to emerge as microbiome research continues to expand into oncological research. It is our hope that this review provides new insights for further CRC and *Fusobacterium* research.

## Author contributions

ZZ conceived and wrote the paper. HH, HY, and JC reviewed and edited the manuscript. All authors read and approved the manuscript.

### Conflict of interest statement

The authors declare that the research was conducted in the absence of any commercial or financial relationships that could be construed as a potential conflict of interest.

## Abbreviations

MSI: microsatellite instability
MDSCs: myeloid-derived suppressive cells
TAMs: tumor associated macrophages
TANs: tumor associated neutrophils
TRUC mice: *T-bet*^−/−^ × *Rag2*^−/−^ ulcerative colitis mice
CRC: colorectal cancer
IBD: inflammatory bowel disease
NF-κB: Nuclear factor kappa-light-chain-enhancer of activated B cells
*F. nucleatum*: *Fusobacterium nucleatum*
APC: adenomatous polyposis coli
RIG-1: retinoic acid inducible gene I
Tregs: regulatory T cells
Gr1: granulocyte differentiation antigen 1
CD: cluster of differentiation
ROS: reactive oxygen species
MLH1: human mutL homolog 1
FIP: *Fusobacterium nucleatum* immunosuppressive protein
PCNA: proliferating cell nuclear antigen
TIGIT: T cell immunoreceptor with Ig and ITIM domains
NK cell: natural killer cell
COX2: cyclo-oxygenase-2
IL: interleukin
TNF: tumor necrosis factor
MMP3: matrix metalloproteinase-3
TLR: toll-like receptor
RASA1: RASp21 GTPase activating protein
RAS: a class of small G-protein
RAF: rapidly accelerated fibrosarcoma
MEK: mitogen-activated protein kinase-ERK kinase
ERK: extracellular regulated mitogen-activated protein kinase
*TP53*: tumor protein *p53*
CHD: chromodomain helicase DNA binding protein
CIMP: CpG island methylator phenotype
GI tract: gastrointestinal tract
MMR: mismatch repair
PGE2: prostaglandin E2
MYD88: myeloid differentiation primary response gene 88
ULK1: unc-51 like autophagy activating kinase 1
ATG7: autophagy-related protein 7
ECM: extracellular matrix
VEGF: vascular endothelial growth factor
PD-1: programmed cell death 1
Th17 cells: T helper 17 cells.
